# ‘Off the shelf’ immunotherapies: Generation and application of pluripotent stem cell‐derived immune cells

**DOI:** 10.1111/cpr.13425

**Published:** 2023-03-01

**Authors:** Chenxin Wang, Jingjing Liu, Wei Li

**Affiliations:** ^1^ State Key Laboratory of Stem Cell and Reproductive Biology Institute of Zoology, Chinese Academy of Sciences Beijing China; ^2^ Institute for Stem Cell and Regenerative Medicine Chinese Academy of Sciences Beijing China; ^3^ Bejing Institute for Stem Cell and Regenerative Medicine Beijing China; ^4^ University of Chinese Academy of Sciences Beijing China

## Abstract

In recent years, great strides have been made toward the development of immune cell‐based therapies in the treatment of refractory malignancies. Primary T cells and NK cells armed with chimeric antigen receptors have achieved tremendous clinical success especially in patients with leukaemia and lymphoma. However, the autologous origin of these effector cells means that a single batch of laboriously engineered cells treats only a certain patient, leading to high cost, ununiform product quality, and risk of delay in treatment, and therefore results in restricted accessibility of these therapies to the overwhelming majority of the patients. Addressing these tricky obstacles calls for the development of universal immune cell products that can be provided ‘off the shelf’ in a large amount. Pluripotent stem cells (PSCs), owing to their unique capacity of self‐renewal and the potential of multi‐lineage differentiation, offer an unlimited cell source to generate uniform and scalable engineered immune cells. This review discusses the major advances in the development of PSC‐derived immune cell differentiation approaches and their therapeutic potential in treating both hematologic malignancies and solid tumours. We also consider the potency of PSC‐derived immune cells as an alternative therapeutic strategy for other diseases, such as autoimmune diseases, fibrosis, infections, et al.

## Graphical abstract

PSCs undergo unlimited self‐renewal and have the potential to differentiate into all cell types, which provides a reliable source of effective immune cells. These PSC‐derived immune cells elicit various immune responses and can be used to treat various diseases, such as leukaemia, solid tumours, autoimmune diseases, respiratory infections, fibrosis, et al.

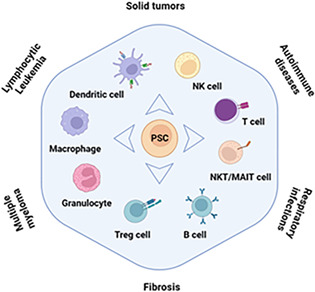

## 1 INTRODUCTION

As a targeted therapeutic approach, immune cell therapy is in rapid development and has become a promising treatment modality with the potential to cure a wide range of diseases.^1^, ^2^, ^3^ At present, nearly a dozen of immune cell therapies have been approved worldwide, most of which are for cancer patients. Notably, all these approved drugs are autologous products, derived from the patient's own peripheral blood. Although peripheral blood cells are readily available and the immune rejection responses of these engineered cells are minimal, the disadvantages are obvious: engineering of patient‐derived immune cells results in high heterogeneity, high cost, and long manufacturing time, which ultimately leads to restricted patient accessibility. To this end, several groups are developing allogeneic immune cell products derived from peripheral blood of healthy donors. In this way, a single batch of drugs can be distributed to a number of patients. However, the expansion capacity of these allogeneic immune cells is still limited. Moreover, to avoid host‐versus‐graft (HvG) reaction and graft‐versus‐host disease (GvHD), primary immune cells generally need to be genetically modified to knock out HLA molecules and, in the case of T cells, to disrupt TCR. In addition, in the treatment of diseases such as cancer, genetic modification for integration of the CAR transgene is often required to improve the function of immune cells.^4^ These genetic modifications in primary cells might increase the risk of tumorigenesis because of a lack of safety certification after genome editing and moreover, might reduce the yield of the final product because of the limited editing efficiencies.

**FIGURE 1:**
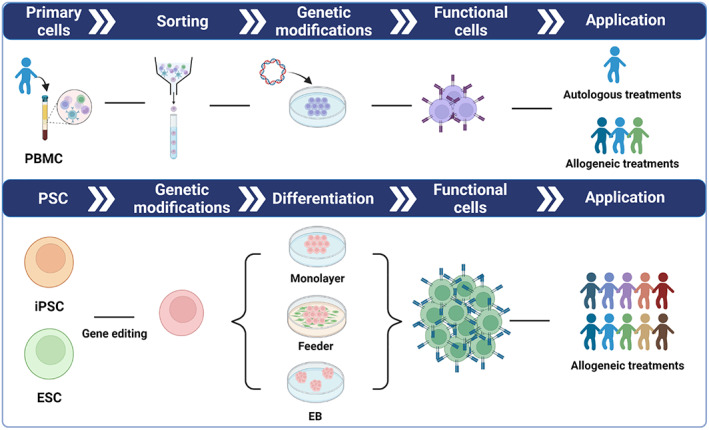
Schematic model of strategies and platforms for generation of peripheral blood‐derived and PSC‐derived immune cells. Based on the unique advantages of PSCs, multiple genetic modifications can be introduced to produce safe, versatile, abundant and functionally enhanced immune cells for clinical applications.

Pluripotent stem cells (PSCs), whether embryonic stem cells (ESCs)^5^, ^6^ or induced pluripotent stem cells (iPSCs),^7^ have unlimited self‐renewal capacity and can be differentiated into various types of immune cells, thus provide an ideal cell source for developing ‘off‐the‐shelf’ cell therapies. Moreover, PSCs provide a convenient platform for genetic modifications at the starting stage, as properly edited cells can be isolated and identified from individual clones and evaluated for off‐target genomic alterations through whole‐genome sequencing. Once certified, a single genetically modified PSC clone can be expanded and differentiated into engineered functional immune cells at a nearly unlimited scale.^8^, ^9^ With the combination of the PSC techniques, efficient lineage‐specific immune cell differentiation approaches, and safe and efficient gene editing methods, PSCs offer an alternative accessible platform to produce engineered immune cells in large amount (Figure 1). At present, approaches for the manufacturing of multiple PSC‐derived immune cells that can be scaled up without serum or feeder cells have also been established. A variety of PSC‐derived immunotherapeutic products have also entered clinical trials.^10^ In this review, we summarize the advances in immune cell differentiation methods (Table 1) and clinical applications of these engineered immune cells derived from human PSCs (Table 2).

**TABLE 1:** Representative differentiation methods for generating PSC‐derived immune cells.

Generated cell type	Cell source	Methods	Serum/feeder status	Total days required	Factors in culture medium	Functional test	Publication
T lineage cell	hESC	Feeder	Serum‐containing Feeder: MEF, OP9, OP9‐DL1	43–61 days	FLT3‐L, IL‐7, SCF	In vitro TCR rearrangement and cytokine production assays	^11^
T lineage cell	hiPSC	Feeder	Serum‐containing Feeder: C3H10T1/2, OP9‐DLL1	16–45 days	VEGF, SCF, FLT3L, IL‐7, IL‐15	In vitro cell cytotoxicity assays	^12^
T lineage cell	hiPSC	Feeder	Serum‐containing Feeder: MEF, OP9, OP9‐DLL1	~40 days	FLT3‐L, IL‐7, SCF, IL‐2, anti‐human CD3, anti‐human CD28	In vitro TCR rearrangement and cytokine production assays	^13^
T lineage cell	hiPSC	EB and DL4‐μbeads	Serum‐free Feeder‐free	~50 days	BMP4, bFGF, SB431542, CHIR99021, IL‐6, IL‐11, VEGF, IGF‐1, SCF, EPO	In vivo thymus engraftment assay	^14^
T lineage cell	hiPSC	EB and immobilized DL4 protein	Serum‐free Feeder‐free	35–42 days	VEGF, BMP4, bFGF, SCF, TPO, FLT3L, SB431542, CHIR99021, IL‐7, OKT3	In vitro and in vivo cell cytotoxicity assays	^15^
T lineage cell	hESC hiPSC	Feeder and artificial thymic organoid (ATO)	Serum‐free Feeder: MS5‐hDLL1/4	67 days	Activin A, BMP4, VEGF, FGF, SCF, FLT3L, TPO, IL‐7, Ascorbic Acid	In vitro TCR rearrangement and cell cytotoxicity assays	^16^
NKT cell	hiPSC	Feeder	Serum‐containing Feeder: C3H10T1/2, OP9‐DLL1	35 days	VEGF, FLT3L, SCF, IL‐7, IL‐2, IL‐15	In vitro cell cytotoxicity assay	^17^
NK cell	hESC	Feeder	Serum‐containing Feeder: MEF, S17, AFT024	21–67 days	IL‐15, IL‐3, IL‐7, SCF, FLT3L,	In vitro cell cytotoxicity and cytokine production assays	^18^
NK cell	hESC hiPSC	EB	Serum‐free Feeder‐free	39 days	VEGF, BMP4, IL‐3, IL‐7, IL‐15, SCF, FLT3L	In vitro specific killing assay	^19^
NK cell	hESC hiPSC	EB	Serum‐free Feeder‐free	34 days	VEGF, BMP4, IL‐3, IL‐7, IL‐15, SCF, FLT3L	In vitro specific killing assay	^20^
NK cell	hESC hiPSC	Feeder and organoid	Serum‐free Feeder: OP9, OP9‐DLL1/DLL4	27 days	BMP4, ACTIVIN A, bFGF, CHIR99021, PIK‐90, A‐83‐01, C59, SB431542, Hydrocortisone, SGR‐SM, FLT3L, TPO, SCF, EGF, VEGF, IGF‐1, Ascorbic Acid	In vitro and in vivo cell cytotoxicity assays	^21^
Macrophage	hESC	EB and Feeder	Serum‐containing Feeder: S17	26–32 days	GM‐CSF, M‐CSF	In vitro phagocytosis, HIV infection, and cytokine production assays	^22^
Macrophage	hESC	EB	Serum‐containing Feeder: MEF	~18 days for first harvest, multiple harvests	M‐CSF, IL‐3	In vitro endocytosis, phagocytosis, cytokine production, and polarization assays	^23^
Macrophage	hESC hiPSC	EB	Serum‐free Feeder‐free	~21 days for first harvest, multiple harvests	BMP4, VEGF, SCF, M‐CSF, IL‐3	In vitro phagocytosis, HIV infection, and cytokine production assays	^24^
Macrophage	hiPSC	EB	Serum‐free Feeder: MEF	~20 days for first harvest, multiple harvests	M‐CSF, IL‐3	In vitro phagocytosis assay and in vivo bacterial infections	^25^, ^26^
Macrophage	hESC hiPSC	Monolayer	Serum‐free Feeder‐free	16–28 days	BMP4, VEGF, SCF, bFGF, SCF, FL3, IL‐3, TPO, M‐CSF, GM‐CSF	In vitro cytokine production, chemotaxis, and polarization assays	^27^
Macrophage	hiPSC	Monolayer	Serum‐free Feeder‐free	15 days	BMP4, Activin A, CHIR99021, SB431542, VEGF, bFGF, SCF, IL‐3, IL‐6, TPO, M‐CSF	In vitro microfluidic flow adhesion, endocytosis, phagocytosis, polarization, and cytokine production and chemokines assays	^28^, ^29^
Dendritic cell	hESC	Feeder	Serum‐containing Feeder: OP9‐DL1	24–29 days	GM‐CSF, IL‐4	In vitro antigen presentation assay	^30^
Dendritic cell	hESC	EB	Serum‐free Feeder‐free	17–25 days	BMP4, VEGF, SCF, GM‐CSF, IL‐4	In vitro phagocytosis and antigen presentation assays	^31^
Dendritic cell	hiPSC	Feeder	Serum‐free Feeder‐free	28 days	GM‐CSF, FLT3L, SCF, VEGF, BMP4	In vitro phagocytosis, antigen presentation, and cytokine production assays	^32^
B cell	hiPSC	Feeder	Serum‐containing Feeder: OP9, MS5	21–42 days	IL‐3, IL‐7, SCF, FLT3L	In vitro VDJ rearrangement assay	^33^
B cell	hESC hiPSC	Feeder	Serum‐containing Feeder: OP9, MS5	39 days	bFGF, IL‐3, FLT3L, SCF, IL‐7	N/A	^34^
Microglia	hESC hiPSC	EB	Serum‐free Feeder: MEF	74 days	M‐CSF, IL‐34	In vitro cytokine production assay	^35^
Microglia	hiPSC	Monolayer	Serum‐free Feeder‐free	26 days	BMP4, VEGF, CHIR99021, FGF2, SCF, DKK1, IL‐3, IL‐6, M‐CSF	In vitro phagocytosis and cytokine production assays	^36^

**TABLE 2:** Clinical trials of PSC/HSC‐derived immune cells for immunotherapies.

Product	Cell type	Genetic engineering target	Indication	Phase	Identifier	Sponsor
FT819	iPSC‐T	CD19 CAR, TCR‐ depletion	Lymphoma and leukaemia	Phase I	NCT04629729	Fate Therapeutics
ALLO‐501A	iPSC‐T	CD19 CAR, TCRα/CD52 depletion	B‐cell lymphoma	Phase I/II	NCT04416984	Allogene Therapeutics
ALLO‐605	iPSC‐T	BCMA CAR, TCRα/CD52 depletion	Multiple myeloma	Phase I/II	NCT05000450	Allogene Therapeutics
ALLO‐715	iPSC‐T	BCMA CAR, TCRα/CD52 depletion	Multiple myeloma	Phase I	NCT04093596	Allogene Therapeutics
ALLO‐316	iPSC‐T	CD70 CAR, TCRα/CD52 depletion	Renal cell carcinoma	Phase I	NCT04696731	Allogene Therapeutics
CNTY‐101	iPSC‐NK	CD19 CAR	B‐cell malignancies	Phase I	NCT05336409	Century Therapeutics
CYNK‐101	HSC‐NK	Fc gamma receptor III	Adenocarcinoma	Phase I/IIa	NCT05207722	Celularity Incorporated
CYNK‐001	HSC‐NK	NA	Acute myeloid Leukaemia	Phase I	NCT04310592	Celularity Incorporated
CYNK‐001	HSC‐NK	NA	COVID‐19	Phase I/II	NCT04365101	Celularity Incorporated
CYNK‐001	HSC‐NK	NA	Multiple myeloma	Phase I	NCT04309084	Celularity Incorporated
FT500	iPSC‐NK	NA	advanced solid tumours	Phase I	NCT03841110	Fate Therapeutics
FT516	iPSC‐NK	Fc gamma receptor III	Acute myeloid leukaemia, B‐cell lymphoma	Phase I	NCT04023071	Fate Therapeutics
FT516	iPSC‐NK	Fc gamma receptor III	solid tumours	Phase I	NCT04551885	Fate Therapeutics
FT538	iPSC‐NK	Fc gamma receptor III, IL‐15RF; CD38 depletion	Multiple myeloma	Phase I	NCT04714372	Fate Therapeutics
FT538	iPSC‐NK	Fc gamma receptor III, IL‐15RF; CD38 depletion	solid tumours	Phase I	NCT05069935	Fate Therapeutics
FT596	iPSC‐NK	Fc gamma receptor III, IL‐15RF, CD 19 CAR	B‐cell lymphoma	Phase I	NCT04245722	Fate Therapeutics
FT536	iPSC‐NK	Fc gamma receptor III, IL‐15RF; CD38 depletion, MICA/B CAR	Advanced solid tumours	Phase I	NCT05395052	Fate Therapeutics
FT573	iPSC‐NK	Fc gamma receptor III, IL‐15RF; CD38 depletion, B7‐H3 CAR	Multiple myeloma	Phase I	NCT05182073	Fate Therapeutics

## 2 PSC‐DERIVED T CELLS

Although the application of engineered primary T cells has achieved great success in the clinic, PSC‐derived T cells are faced with additional challenges because T cell development involves TCR rearrangement and positive/negative selection in vivo. Generation of PSC‐derived T cells involves a complicated process and requires two essential stages: PSCs are differentiated into haematopoietic progenitor cells or haematopoietic stem cells (HPCs/HSCs) in the first stage and then, HPCs/HSCs are committed into T cell lineages via Notch signalling in the second stage.^37^ Galic demonstrated that human ESCs can be specified into a T lymphoid lineage by coculture with feeder cells and subsequent engraftment into thymic tissues in immunodeficient mice.^38^, ^39^ However, these T cells were limited in cell number and their functions were not evaluated. Mouse iPSC‐derived functional T cells were first generated by Lei et al. via coculture with stromal cells OP9‐DL1.^40^ These iPSC‐derived T cells secreted IL‐2 and IFNγ after activation and restored the T‐cell pool in *Rag1*‐deficient mice. In 2014, Kishino generated transgene‐free human peripheral blood T cell‐derived iPSCs in a defined culture medium and a feeder‐free condition, making them more suitable for therapeutic applications.^41^ In a more recent study, iPSC‐derived T cells were demonstrated to be capable of expanding up to 10,000‐fold. Furthermore, these T cells expressed CD8αβ^+^ and exhibited improved antigen‐specific cytotoxicity compared with CD8αα^+^ T cells and prolonged the survival of murine models with tumours.^42^

iPSCs derived from non‐T cells (non‐T iPSCs), such as fibroblasts and primary skin cells, bear unrearranged TCR. After T cell differentiation in vitro, these iPSC‐derived T lymphocytes display a diverse TCR repertoire.^43^ However, since no positive and negative selection occurs, these iPSCs can only be used for studying normal T cell development and disease modelling; they are not suitable for clinical applications considering autoreactivity of T cells.^44^, ^45^ In addition, transduction of specific exogenous TCR receptors in non‐T iPSCs, followed by generation of CD8αβ T cells with antigen‐specific cytotoxic activity comparable to CD8αβ T regenerated from T‐iPSCs, similarly inhibited tumour growth in xenograft tumour models.^46^ In 2013, antigen‐specific T cells derived from iPSCs were generated.^12^, ^13^, ^47^ Nishimura T amplifies antigen‐specific CD8 T cells from HIV‐infected patients and then sub‐induces them into iPSCs (T‐iPSCs). Further re‐differentiation generated CD8 T cells with high proliferative capacity, elongated telomeres, and antigen‐specific killing activity.^12^ Because the *TCR* gene of a T‐iPSC clone has been rearranged to be antigen‐specific, all T cells obtained from differentiation of this iPSC line bear a specific rearranged TCR originated from the T‐iPSC clone that is identical to the original T cell and respond specifically to the original epitope.^41^, ^48^, ^49^, ^50^, ^51^ More recently, Michaels et al. established a serum‐ and feeder‐free differentiation system with the addition of DLL4 and VCAM1 during the endothelial‐to‐haematopoietic transition, which promoted T cell production by approximately 80‐fold.^52^

Furthermore, the 3D‐organoid systems, such as artificial thymic organoid (ATO) and fetal thymic organ culture systems, were established for the generation of mature T cells derived from PSCs.^16^, ^51^, ^53^, ^54^, ^55^, ^56^ In particular, the ATO‐based differentiation system was used to induce T‐iPSCs derived from CD62L^+^ naive and memory T cells into CD8αβ^+^ T cells. These T cells showed comparable antigen‐specific activation, degranulation, cytotoxicity, and cytokine secretion capabilities to conventional engineered primary T cells.^51^

Other strategies have enhanced T cell‐specific lineage commitment via genetic modifications in iPSCs. *EZH1* knockdown facilitates differentiation and maturation of T cells from iPSCs in vitro, displaying a highly diverse TCR repertoire and mature molecular signatures similar to TCRαβ T cells from peripheral blood.^57^ Genetic modification of the *FoxP3* gene in iPSC cells and stimulation with Notch ligand in vitro direct iPSC differentiation into regulatory T cells (Tregs).^58^ Furthermore, Haque retrovirally transduced murine iPSCs with specific *TCR* and *FoxP3* and developed an Ag‐specific Treg with the ability to suppress autoimmunity in a murine model of arthritis.^59^

In addition to cytotoxic T cells, PSCs can also differentiate into unconventional T cells, including natural killer T (NKT), MAIT, and gamma delta T (γ δ T) cells. Watarai generated ESC lines by reprogramming and rearranging T cell receptor gene using the nuclear transplantation technique. These ESCs were induced to differentiate into NKT2 under specific conditions and in the presence of Notch signalling and into NKT1 in the absence of Notch signalling.^60^ In addition, several groups confirmed that induction of iPSCs with reprogramming factors from mature NKT is feasible.^17^, ^61^ These iPSCs can differentiate into NKT1 cells, which secrete IFNγ cytokine and poss an anti‐tumour activity. MAIT can also be reprogrammed into iPSCs and then re‐differentiated into MAIT‐like cells. These iPSC‐derived MAIT expressed MAIT‐related markers, secreted cytokines, and had immune activity in vitro and in vivo.^62^ In addition, γ δ T cells can also be derived from γ δ T‐iPSCs in vitro.^63^

## 3 PSC‐DERIVED NK CELLS

Current NK cell immunotherapies come from a variety of cell sources in vitro, including the peripheral blood mononuclear cells (PBMCs), NK‐92 cell line, umbilical cord blood (UCB), and PSCs.^9^, ^64^, ^65^ Although PBMCs and UCB are readily available and can be expanded in vitro, yield and function are influenced by donor heterogeneity and depend on the purity of the starting NK cells. NK‐92 cell line has good homogeneity and can be easily obtained and genetically modified. But its proliferation needs to be inhibited by irradiation or other means before administration to minimize the risk of tumorigenesis, which might affect its anti‐tumour activity.

A few research groups have successfully differentiated PSCs into homogeneous NK cells that are similar in phenotype and effector function when compared with primary NK cells.^66^, ^67^ Similar to PSC‐derived T cells, the differentiation of PSC‐derived NK cells can also be divided into two stages. First, PSCs need appropriate signals to be committed toward HSCs/HPCs, followed by further differentiation of HSCs/HPCs into NK cells. The process of this two‐stage differentiation strategy takes approximately 1–2 months. PSC‐derived NK cells are over 90% pure and have similar growth rates, phenotypes, and functions to PBMCs and UCB‐derived NK cells.^64^

Based on differentiation strategies, generation of PSC‐derived NK cells can be divided into 2D and 3D culture systems. Although hESC‐derived NK cells generated based on coculture with feeder cells in a 2D differentiation system were identified earlier,^68^, ^69^ Kaufman's group was the first to conduct an in‐depth analysis of the phenotype and function of hESC‐NK cells, and the results showed that these hESC‐derived NK cells had a mature phenotype: they secreted cytokines and were toxic to tumour cells in vivo and in vitro.^18^, ^70^ iPSCs have also been successfully differentiated into NK cells with potent antiviral activity and anti‐tumour activity.^71^, ^72^, ^73^ However, these culture systems rely on the use of mouse stromal cells, which are prone to a safety risk of cross‐species contamination. To eliminate the use of stromal cells and serum, and to simulate the natural developmental cues of early‐stage embryos, a 3D culture system for human PSC‐NK has emerged.^19^, ^74^, ^75^ Spin embryoid body (EB)‐based induction method differentiates PSCs into HPCs without sorting or mouse stromal cell support, and the HPCs are further differentiated into NK cells in the presence of IL‐3, IL‐7, IL‐15, SCF, and Flt3L cytokines. This system not only provides a source of mature, effective, and cytotoxic NK cells but also a method for clinical‐scale expansion of anti‐tumour lymphocytes.^19^, ^76^ Recently, Huang et al. established an organoid aggregation‐based method that combined lateral plate mesoderm cells and OP9 feeder cells and achieved a thousand‐fold expansion of PSC‐derived NK cells (single hPSC yielded more than 1000 NK cells).^21^

### 3.1 PSC‐derived macrophages

Due to the limited number of methods available for purification and expansion of human macrophages, various approaches have been developed to generate macrophages from PSCs. The advantages of PSC‐derived macrophages include good scalability, high standardization, unlimited quantities, better modelling of tissue‐resident macrophages (TRM), and ease of genetic modifications.^77^, ^78^, ^79^ Meanwhile, macrophages derived from disease‐specific iPSCs provide a useful platform for elucidating disease pathogenesis, analysing the association of genetic mutations with disease, and screening new drug candidates.^79^, ^80^

The differentiation strategies of macrophages from PSCs mainly include co‐culture systems based on feeder cells, culture systems based on 3D aggregate structure, and feeder‐free monolayer differentiation systems. The co‐culture protocols comprise two major steps: induction of HPC differentiation by coculture of PSCs with feeder cells and directed differentiation of HPCs into macrophages.^22^, ^30^, ^81^, ^82^, ^83^, ^84^ Slukvin's group demonstrated for the first time the feasibility of using iPSCs to differentiate into macrophages.^82^ Progenitor cells can also be expanded briefly before macrophage differentiation.^85^ In 2008, an EB‐based differentiation method was reported for producing homogeneous monocyte‐like cells from hESCs, which is simple and efficient and does not require additional purification.^23^ A few groups have optimized EB‐based method for macrophages differentiation from human PSCs.^25^, ^86^, ^87^, ^88^, ^89^, ^90^ For example, Van Wilgenburg described a serum‐ and feeder‐free culture condition that produced very consistent, pure, and high‐yield monocytes and macrophages from human ESCs via EB.^24^ A feeder‐free, chemically defined monolayer culture system has been developed in which cells are maintained on tissue culture dishes coated with growth factor‐reduced Matrigel.^27^ Other groups have recently reported on the scheme of monolayer differentiation strategies.^28^, ^36^, ^91^, ^92^ However, due to the required purification steps and/or adhesion‐based culture conditions, these protocols do not allow the generation of clinically relevant quantities of iPSC‐derived cells. Lachmann's group reported a technology based on a 3D suspended stirred tank bioreactor that serves as a scalable and stable production platform to deliver large‐quantity, highly pure, and functional iPSC‐derived macrophages.^25^, ^26^, ^93^ In addition, regarding to the storage of PSC‐derived macrophages, several recent publications describe effective cryopreservation such as CS10.^28^, ^29^ Alternatively, cells can be stored in a spinner flask for short‐term storage.^25^, ^94^

It has long been clear that macrophages in tissues are heterogeneous in origin and have acquired tissue‐specific functions and characteristics.^95^ These TRMs play critical roles in tissue development, maintenance homeostasis, tissue regeneration, and repair, whereas studying human TRMs is challenging due to the lack of appropriate cells from human fetal or adult tissues for comprehensive in vitro studies.^77^ Therefore, PSCs are a renewable source of TRM. Various protocols have been developed to generate functional TRMs from PSCs via restating appropriate molecular signals in vitro and re‐creation of the events and specific environments that occur during development of TAM in vivo, such as microglial,^35^, ^96^, ^97^, ^98^, ^99^, ^100^, ^101^ alveolar macrophages,^102^, ^103^ and so on.^36^ In addition, several studies have established novel approaches for the rapid conversion of PSCs into functional TRMs by overexpressing lineage‐specific transcription factors, such as SPI1 and/or CEBPA.^104^, ^105^

## 4 OTHER IMMUNE CELL TYPES DERIVED FROM PSCS

Dendritic cells (DCs) are a group of functionally specialized myeloid‐origin antigen‐presenting cells that play a critical role in bridging the gap between innate and adaptive immunities and maintaining tolerance.^106^, ^107^ DCs play a key role in activating antigen‐specific T cells, initiating anti‐tumour immune response, and maintaining immune tolerance in specific antigen‐presenting environments. Thus, they are potential therapeutic targets for cancer immunotherapies to generate a robust immune response and improve anti‐tumour cytotoxicity.^108^, ^109^, ^110^, ^111^ Differentiation protocols for PSC‐derived DCs were developed in the past two decades. Slukvin et al.^30^ generated PSC‐derived DCs whose phenotype and function were comparable with DCs differentiated from bone marrow CD34^+^ haematopoietic progenitor cells.^30^ In 2009, Tseng developed a serum‐ and feeder‐free culture system to generate functional human ESC‐derived DCs that phagocytose, process, and present antigen upon maturation, demonstrating the potential of PSC‐derived DCs in immunotherapy.^31^ Thereafter, several groups have also optimized the protocols for DC differentiation from PSCs.^82^, ^84^, ^112^, ^113^, ^114^, ^115^ Sachamitr reported that they were able to successfully differentiate DCs with tolerance maintenance properties from PSCs through the spontaneous formation of embryoid bodies and exposure to a cocktail of growth factors.^116^ However, recent evidence suggests that DC‐like cells derived from human iPSCs are heterogeneous and include various subsets such as conventional type 1 DC (DC1), DC2, DC3, AS‐DC and so on, but not plasmacytoid DCs.^32^

In addition, PSCs can also be differentiated into B cells and granulocytes. The approaches of B cells generated from human PSC‐derived haematopoietic progenitor cells have been developed over the past several years, and these B cells are able to undergo VDJ rearrangement and express immunoglobins, such as IgM as well.^33^, ^34^, ^68^, ^117^, ^118^ Böiers characterized B progenitor cells produced by in vitro differentiation of human PSCs that were similar to B cells in early fetal liver.^117^ Recently, Zhang et al. established a de novo approach to specifically generate the entire lineage of B cells, including innate B‐1a, B‐1b, marginal zone B cells, and adaptive follicular B cells, by forced expression of transcription factors, such as *Lhx2*, *Hoxa9*, and *Runx1* in mouse ESCs.^119^ Neutrophils derived from PSCs have relatively conserved functions, including phagocytosis, migration via cytokine, respiratory burst response, and induction of neutrophil extracellular traps.^82^, ^85^, ^93^, ^120^, ^121^, ^122^ PSC‐derived eosinophils display eosinophil‐specific characteristics comparable to primary naive eosinophils, which are highly cell cytotoxic and inhibit tumour growth in vivo.^123^

## 5 PSC‐DERIVED IMMUNE CELLS FOR CANCER THERAPY

Chimeric antigen receptor T cells have achieved tremendous clinical success in treating refractory malignancies, such as leukaemia. The first generation of CAR is a fusion of a single‐chain variable fragment (scFv), a transmembrane domain, and the intracellular signalling domain derived from the CD3ζ molecule. The scFv module is derived from an antibody recognizing a specific antigen presented by cancer cells, taking CD19 as an example.^124^, ^125^ The CD3ζ signalling molecule contains three immunoreceptor tyrosine‐based activation motifs (ITAMs) that are stimulated upon phosphorylation.^126^, ^127^ To enhance the tumour‐killing efficacy of CAR T cells, a co‐stimulatory domain, derived from either CD28 or 4‐1BB, is inserted between the transmembrane and CD3ζ signalling domains to form the second‐generation CARs.^128^, ^129^ Till now, all current FDA‐approved CAR T products follow a second‐generation CAR design with either a CD28 or 4‐1BB co‐stimulatory domain.^130^, ^131^ The third generation of CARs integrates two co‐stimulation domains in one CAR construct. In addition to the most commonly used CD28 and 4‐1BB co‐stimulatory modules, other co‐stimulatory domains used in CAR design are derived from CD27,^132^ OX40,^133^ and inducible T cell co‐stimulator (ICOS).^134^ The fourth generation of CARs, also referred to as armoured CARs, produces an additional protein molecule, such as cytokines for tuning of CAR signalling to enhance proliferation and persistence of CAR T cells in the body.^135^, ^136^ To date, the United States Food and Drug Administration (FDA) has approved six CAR T cell products for the treatment of relapsed refractory B‐cell malignancy or multiple myeloma. Notably, all these cells are patient‐derived autologous products, reflecting that CAR T drugs are complex, costly, individualized therapies and more cost‐effective, ‘off the shelf’ immune cell products are urgently in need. Solid tumours account for more than 90% of human malignancies and are largely refractory to engineered immune cell‐based therapies, mainly because of the low rate of immune cell infiltration and the immunosuppressive tumour microenvironment (TME). Nonetheless, tremendous efforts have been taken to conquer the obstacles and it is optimistic to expect that effective immunotherapeutic approaches for common solid tumours will emerge in the near future. Such a prospective advancement calls for unlimited numbers of homogeneous, standardized immune cell products engineered to ensure safety, potency, and reproducibility of cell‐based anti‐tumour immunotherapies.

PSCs are flexible for large‐scale expansion and most importantly, are amenable to complex genetic modifications thus conferring designed properties, such as tumour targeting specificity, persistence in the immune suppressive environment, release of certain cytokines, and self‐elimination program, to the PSC‐derived immune products via lineage‐specific differentiation approaches. Considering that CAR T cells have achieved profound responses in treating patients with B cell lymphoma in the clinic, it is logical to first explore the anti‐tumour efficacy of PSC‐derived CAR T cells. As described above, the generation of PSC‐derived T cells varies in both efficiency and cell functions, making intractable challenges for clinical applications of these cells.^12^, ^13^, ^46^, ^47^, ^137^ Another obstacle imposed to the application of PSC‐derived CAR T cells is the occurrence of random TCR rearrangement during T lymphocyte maturation, which might result in unpredictable alloreactivities. One way to solve this problem is the application of T‐iPSCs as the starting material for lineage‐specific differentiation, as T‐iPSC‐derived T cells retain the endogenous TCR rearrangement.^13^ In 2013, Themeli et al. reported the first T‐iPSC‐derived CAR T cells. A CD19‐specific CAR was integrated into one of the iPSC clones by lentiviral transduction, the engineered iPSCs were then differentiated into T cells following a feeder cell coculture protocol. The T‐iPSC‐derived T cells expressed the CD19‐specific CAR, and its anti‐tumour potential was demonstrated by coculture with CD19‐expressing cells in vitro and in tumour xenograft models.^47^ However, the anti‐tumour efficacy of these early iPSC‐derived CAR T cells was not a patch on the engineered primary T cells, mostly because of the lack of CD4^+^ T cells in iPSC‐derived CAR T products.^15^ Recently, Cui et al. developed a method for complete maturation of PSC‐derived T lymphocytes that functionally resemble natural T cells. These T cells were infected with CD19‐CAR retrovirus to generate CD19‐CAR iT cells. They showed that these CD19‐CAR iT cells can significantly proliferate and effectively eradicate B lymphoma cells in tumour‐bearing mice, demonstrating promising therapeutic potential for treating blood cancers.^138^ T‐iPSCs derived from a tumour antigen‐specific T cell clone were also utilized for production of cytotoxic T cells with their original anti‐tumour TCR.^42^, ^46^ Moreover, it was reported that the T‐iPSCs might retain epigenetic memories of their T lymphocyte origin that can be harnessed for more determined lineage specification.^139^ Alternatively, by inactivation of the RAG recombinase gene^46^ or inserting a CAR in the *TCRA* locus (encoding TCRα)^140^ with genome editing approaches, non‐T cell‐derived iPSCs could also be used for production of a bank of engineered CAR T cells. Another constraint to the therapeutic application of iPSC‐derived CAR T products is the HvG immune rejection effect. Considering the highly polymorphic nature of the HLA system that dominants in HvG, construction of a bank of iPSCs with homozygous HLAs might simplify the process of HLA matching.^141^, ^142^ Alternatively, Wang et al. exploited genome‐editing technologies to delete the genes encoding an MHC‐I subunit (*B2M*), a transcription regulator of MHC‐II (*CIITA*), and an NK cell ligand poliovirus receptor CD155, accompanied by integration of the protective NK cell‐inhibitory ligand HLA‐E, and finally generated ‘invisible iPSC‐derived T cells and NK cells’ that minimized recognition by the host immune system.^143^

Clinical trials of iPSC‐derived CAR T cells are in progress and the Fate Therapeutics company is a pioneer in this field. Fate Therapeutics have developed a feeder‐free differentiation protocol to generate scalable iPSC‐derived CAR T cells. In the FT819 CAR T product, a CD19 CAR was integrated into the *TCRα* locus to obtain regulated CAR expression and to overcome GvHD effect. The CD3ζ module of the CAR construct was also modified to achieve prolonged persistence of the engineered CAR T cells.^126^ When transferred into an in vivo xenograft model, FT819 exhibited enhanced anti‐tumour efficiency and extended survival rate when compared with sham control and primary CAR T cells with the same CD19 CAR.^144^ Recently, a Phase I dose‐finding study of FT819 as monotherapy and in combination with IL‐2 in subjects with relapsed/refractory B‐cell Lymphoma, Chronic Lymphocytic Leukaemia, and Precursor B‐cell Acute Lymphoblastic Leukaemia is in progress (NCT04629729). In 2019, The Center for iPS Cell Research and Application (CiRA) at Kyoto University transferred a novel induced pluripotent stem (iPS) cell‐derived chimeric antigen receptor (CAR) T‐cell therapy (iCART) to Takeda Pharmaceutical to promote clinical testing and commercialization of the iCART product. Recently, Allogene Therapeutics initiated its first allogeneic CAR T Phase II trial, to evaluate its allogeneic iPSC‐derived CAR T product, ALLO‐501A, in Relapsed/Refractory Large B Cell Lymphoma (NCT04416984). ALLO‐501A is engineered to express a CD19 CAR, accompanied by *TCRα* and *CD52* depletion with TALEN, a highly efficient gene editing technology. Deletion of the *CD52* gene in this AlloCAR T product allowed for simultaneous administration of ALLO‐647, an anti‐CD52 mono‐antibody, to eliminate host immune cells selectively and temporarily. ALLO‐605 is a next‐generation AlloCAR T investigational therapy that targets the B‐cell maturation antigen (BCMA) for the treatment of patients with relapsed/refractory multiple myeloma and other BCMA‐positive malignancies, which incorporates Allogene's proprietary TurboCAR technology to allow for cytokine activation signalling to be engineered selectively into CAR T cells. Based on the potential of ALLO‐605 to address the unmet need for patients who have failed other standard multiple myeloma therapies, the United States FDA granted Fast Track designation to ALLO‐605, and the Phase I dose escalation portion evaluating ALLO‐605 was initiated in 2021 (NCT05000450). Other iPSC‐derived CAR T product pipelines of the Allogene Therapeutics company, such as ALLO‐715 and ALLO‐316 are also understudies of Phase I clinical trials (NCT04093596 and NCT04696731).

NK cells process multiple cytotoxicity pathways and are capable of regulating immune responses through production of various cytokines. There is a complex array of activating and suppressive receptors located on the surface of NK cells that distinguish between healthy and ‘stressed’ cells (such as virally infected cells or tumour cells).^145^ Upon forming immunological connections with a target, the behaviour of an NK cell is determined by the cumulative signal generated from engaged receptors toward ligands on the target cell.^146^ Once the target cell recognition is complete, NK cells elicit a ‘killing’ response through the release of cytolytic granules and cytotoxic cytokines.^147^ Moreover, they may also conduct antibody‐dependent cellular cytotoxicity (ADCC) when their CD16 receptor is engaged with antibody‐coated cells.^148^ NK cells play a pivotal role in anti‐cancer immunity and have been used as immunotherapeutic agents in cancer therapies. Given the limited numbers of primary NK cells that can be purified from autologous sources, differentiation of NK cells from PSCs provides approaches for large‐scale production of these cells for immuno‐therapeutic applications. While generation of functional PSC‐derived T cells is indeed inefficient, protocols for NK cell production from PSCs are now much more routinely developed.^19^, ^73^, ^75^, ^149^ Unlike the therapeutic T cell products that recognized non‐self‐host cells via TCR, NK cells recognize their targets in a human leukocyte antigen (HLA)‐unrestricted manner and thus do not present GvHD risks,^150^ making allogeneic NK cells derived from PSCs to be attractive candidates for universal cellular immunotherapies.^151^, ^152^ In addition to the requirement of a high‐efficiency differentiation method, gene‐editing strategies were also introduced in PSC‐derived NK cell manufacturing to enhance their anti‐tumour efficacy, proliferation, and persistence in the tumour environment.

A few reports have demonstrated the anti‐tumour potencies of iPSC‐derived NK cells in preclinical studies. For example, Cichocki et al. established that NK cells derived from iPSCs functioned not only in prohibition of hematologic malignancies but also in controlling the growth of solid tumours. They also revealed that transport of iPSC‐derived NK cells accompanied by PD1 blockade could benefit local T cell infiltration and activation in the immunosuppressive TME.^71^ It was reported that NK cell‐mediated ADCC was attenuated because of the downregulation of the high affinity 156 V variant of CD16a on the surface of activated NK cells by a disintegrin and metalloprotease‐17.^153^ To this end, Zhu et al. generated a non‐cleavable derivative (hnCD16a) by introduction of an S197P mutation at the cleavage site and integrated the receptor mutant into iPSCs, they thus developed hnCD16a‐iPSC‐NK cells that stably expressed CD16a and mediated improved ADCC in pre‐clinical models.^154^ In the clinic, Fate Therapeutics have developed a variety of universal, off‐the‐shelf NK products as therapeutic candidates for clinical investigations. FT500 is the first‐ever iPSC‐derived cell therapy cleared for clinical investigation and approved by the United States FDA. The product is now being investigated in an open‐label, multi‐dose Phase I clinical trial for the treatment of advanced solid tumours (NCT03841110). Recently posted results of this Phase I clinical trial demonstrated that when combined with low‐dose IL‐2 and concurrent ICI therapy, FT500, the first iPSC‐derived NK cell product to be administered in patients with advanced relapsed/refractory solid tumours, is safe and tolerable, with evidence of durable anti‐tumour activity observed in heavily pre‐treated patients resistant to anti‐PD‐1/PD‐L1 therapy (https://fatetherapeutics.com/pipeline/immuno‐oncology‐candidates/ft500/). FT516 is another universal, off‐the‐shelf natural killer (NK) cell cancer immunotherapy derived from a clonal master iPSC line engineered to express the non‐cleavable CD16 (hnCD16) Fc receptor, which is now being investigated in an open‐label, multi‐dose Phase I clinical trial as a monotherapy for the treatment of acute myeloid leukaemia and in combination with CD20‐directed monoclonal antibodies for the treatment of advanced B‐cell lymphoma (NCT04023071). Preliminary clinical results demonstrated objective responses in three of four patients treated with FT516 in combination with rituximab (https://fatetherapeutics.com/pipeline/immuno-oncology-candidates/FT516/). In addition, another FT516 Phase I clinical trial in combination with monoclonal antibody therapy, including PDL1‐, PD1‐, EGFR‐ and HER2‐targeting therapeutic antibodies, across a broad range of solid tumours is in progress (NCT04551885). Recent clinical results showed that of the 12 patients treated with FT516 in combination with Avelumab, the best overall responses of partial response and stable disease were observed in 1 and 6 patients, respectively (https://fatetherapeutics.com/pipeline/immuno-oncology-candidates/FT516/). Besides the hnCD16 Fc receptor, FT538 incorporates two additional functional gene modifications: an IL‐15 receptor fusion (IL‐15RF) that promotes enhanced NK cell activity and the elimination of CD38 expression to mitigate the potential for NK cell fratricide. Phase I clinical trials of FT538 as a monotherapy and in combination with monoclonal antibody therapies for treatment of multiple myeloma and advanced solid tumours are recruiting participants (NCT04714372) (NCT05069935) (NCT04614636).

Fate Therapeutics are also developing a series of iPSC‐derived CAR NK products, such as FT596, FT536, FT573, and FT 576, with FT596 being the most investigated in advance. FT596 harbours the engineered hnCD16a Fc receptor and the IL‐15RF stimulator as described above, and in addition, an NK cell‐modified CD19‐specific CAR (NKG2D transmembrane domain accompanied by a fusion of 2B4 and CD3ζ endodomain). Preclinical research has shown high cytotoxic activity of FT596.^155^ At present, a Phase I study is ongoing to evaluate FT596 as a monotherapy and in combination with rituximab for the treatment of advanced B‐cell lymphoma, or in combination with obinutuzumab for the treatment of CLL (NCT04245722). All of the other three iPSC‐derived NK cell products of this company (FT536, FT573, and FT 576) comprise IL‐15RF, hnCD16a, and CD38 knockout, while containing distinct CAR constructs targeting various tumour‐specific antigens^156^, ^157^ (NCT05182073; NCT05395052). In addition to Fate Therapeutics, other companies such as Cytovia Therapeutics, CellOrigin, and Neukio Biotherapeutics are also focusing on the differentiation, expansion, and genetic engineering of iPSC‐derived NK cells to target tumours.

Macrophages are monocytic immune cells with both a bone marrow (BM)‐derived monocytic origin and a tissue‐specific embryonic origin.^158^, ^159^, ^160^ Tumour‐associated macrophages (TAMs) that infiltrate the tumour tissue are an essential component of the TME and play important roles in constructing and orchestrating the inflammatory cues. Generally, macrophages are plastically polarized into two distinct activation forms in response to diverse environmental signals, which are referred to as M1 (or classic) and M2 (or alternative). M1 macrophages are associated with macrophage‐dependent inflammatory tissue damage and tumour suppression, whereas M2 polarization promotes tissue repair and remodelling and tumour resistance to the adaptive immune system.^161^ As a reflection of the plasticity of their activation forms, TAMs are considered double‐edged swords with dual activating potential in cancer.^162^, ^163^, ^164^, ^165^ However, macrophages resident in TMEs is more frequently to obtain an immunosuppressive M2 polarized phenotype. Nonetheless, macrophages have great potential to be engineered as therapeutic tools in cancer therapy, especially for solid tumours. (They have a natural propensity to traffic into solid tumours.) To this end, macrophages were loaded with various CAR constructs to elicit their anti‐tumour potential. In 2020, Klichinsky et al. transduced a *HER2*‐specific CAR into human macrophages (CAR‐Ms) via an adenoviral vector and demonstrated that a single administration of the engineered CAR‐Ms in xenograft mouse models decreased tumour burden and prolonged overall survival. More importantly, further analysis revealed that the infiltrated CAR‐Ms exhibited a pro‐inflammatory M1 polarization phenotype in the TME.^166^ Based on this study, in 2021, Carisma Therapeutics launched Phase I, first‐in‐human study of the anti‐*HER2* CAR‐M in subjects with *HER2* overexpressing solid tumours (NCT04660929). More recently, Zhang et al. have successfully engineered iPSC‐derived macrophage‐like progenitors and developed CAR‐iMacs, which harboured a CD19‐specific or mesothelin‐specific CAR to enhance their phagocytosis and pro‐inflammatory immune activities.^167^ Right now, CellOrigin Biotech and Qilu Pharmaceutical are collaborating together to push CAR‐iMac pipelines forward to clinical trials (https://www.labiotech.eu/trends‐news/cellorigin‐biotech‐and‐qilu‐pharmaceutical‐to‐develop‐car‐imac‐cell‐therapy/). Besides, companies, such as Myeloid therapeutics, Enlivex, Shoreline Biosciences, and Inceptor Bio are also focusing on the production and engineering of PSC‐derived macrophages to target tumours.

## 6 BEYOND CANCER THERAPY: PSC‐DERIVED IMMUNE CELLS FOR AUTOIMMUNE DISEASES, FIBROSIS, MICROBIAL INFECTIONS, AND IMMUNE REGULATION IN ENGRAFTMENT

Autoimmune diseases (AIDs) such as Type I diabetes (T1D), rheumatoid arthritis, and lupus erythematosus, occur when the immune system goes awry and starts to attack the body's healthy cells. In the past few decades, immunotherapies including biological drugs and small molecule inhibitors targeting inflammatory cytokines, immune cells, and intracellular kinases have become the standard of care to treat AIDs.^168^ In spite of this, engineered immune cells provide a new avenue for the treatment of refractory AIDs. In 2012, Rizwanul Haque et al. reported the differentiation of Treg cells from iPSCs by forced expression of *Foxp3* and coculture on Notch ligand‐expressing stromal cells, they demonstrated that the iPSC‐derived Treg cells significantly suppressed host immune responses and reduced arthritis development within murine models.^58^ The iPSC‐derived Treg cells were also retrovirally transduced with autoantigen‐specific TCRs to generate Ag‐specific iPSC‐Tregs, which exhibited abilities to suppress the development of autoimmune arthritis and T1D after adoptive transfer in murine models.^59^, ^169^ Targeting Ag‐presenting cells, especially the self‐killing infiltrating T cells with engineered CAR T cells is also expected to have therapeutic potential in autoimmune diseases.

In addition to application in cancer therapies, iPSC‐derived macrophages provide a promising avenue for ameliorating fibrosis in degenerative organs and elimination of microbial infections. Somayeh Pouyanfard et al. differentiated human iPSCs into macrophages that exhibit classical surface cell markers and phagocytic activity similar to their peripheral blood‐derived counterparts. Moreover, they demonstrated that these cells were efficiently polarized to pro‐inflammatory M1 or anti‐inflammatory M2 phenotypes in presence of LPS + IFN‐γ and IL‐4 + IL‐13, respectively. They also observed that the M2 iPSC‐derived microphages significantly reduce fibrogenic gene expression and disease‐associated histological markers including Sirius Red, αSMA, and desmin in immunodeficient mice models.^170^ *Staphylococcus aureus* is a common offending organism that causes respiratory infections in the lung. Recent studies have exploited iPSC‐derived macrophages in the treatment of pulmonary *S. aureus* infections. Adoptive transfer of iPSC‐derived macrophages resulted in reduced bacterial load, reduced granulocyte infiltration, and less damage in lung tissue in *S. aureus*‐infected immunodeficient murine models.^25^, ^171^ In addition, PSC‐derived DCs can also participate in the regulation of immune responses. For example, tolerogenic DCs can be produced through genetic modification of DCs. It was reported that DCs derived from CTLA4‐Ig/PD‐L1‐expressing hESCs could maintain immune suppressive properties, induce the activation of Treg cells, and protect allografts from immune rejection in vivo.^172^

## 7 PROSPECTS AND CHALLENGES

Although autologous immune cell therapies, especially the CAR‐engineered primary T lymphocytes in anti‐cancer treatment, have demonstrated promising clinical application potential, the batch‐to‐batch heterogeneity leads to high cost, poor scalability, inadequate quality control, and risk of treatment delay.^173^, ^174^ PSCs undergo unlimited self‐renewal and have the potential to differentiate into all adult tissue types under proper culture conditions.^175^ The PSC technology provides an alternative way for the production of cost‐effective, large‐scale, and uniformly qualified immune cells for treatment of cancers and other diseases via immunotherapies. Moreover, these cells can be readily engineered to upload multiple genetic modifications to ensure the efficacy, specificity and safety of the PSC‐derived immune products. Plenty of clinical investigations on PSC‐derived immune cell therapies have been implemented worldwide. Nonetheless, these technologies are still faced with several obstacles and challenges. Safety is a major concern of the PSC‐based immunotherapies: first of all, contaminating undifferentiated stem cells or precursors might be tumorigenic owing to their pluripotency; second, the complex and time‐consuming manufactory pipeline raises the risk of microbial contamination and thus threaten the quality control of the products. Other remaining issues include the source and qualification of the PSC clones, evaluation of the potency of the PSC‐derived immune cells compared with their autologous counterparts, and influence of the gene editing technologies that are exploited in immune cell engineering on genome stability.

## AUTHOR CONTRIBUTION

CXW and JJL involved in literature collection and manuscript preparation. WL supervised this work.

## CONFLICT OF INTEREST STATEMENT

The authors declare no conflict of interest.

## Data Availability

Data sharing is not applicable to this review as no new data were created or analyzed.
